# A systematic review of the use of an expertise-based randomised controlled trial design

**DOI:** 10.1186/s13063-015-0739-5

**Published:** 2015-05-30

**Authors:** Jonathan A. Cook, Andrew Elders, Charles Boachie, Ted Bassinga, Cynthia Fraser, Doug G. Altman, Isabelle Boutron, Craig R. Ramsay, Graeme S. MacLennan

**Affiliations:** Centre for Statistics in Medicine, Nuffield Department of Orthopaedics, Rheumatology and Musculoskeletal Sciences, Botnar Research Centre, University of Oxford, Nuffield Orthopaedic Centre, Windmill Road, Oxford, OX3 7LD UK; Health Services Research Unit, University of Aberdeen, Health Sciences Building, Foresterhill, Aberdeen, AB25 2ZD UK; Centre d’Epidémiologie Clinique, Université Descartes, Hôpital Hôtel Dieu, 1 Place du Parvis Notre Dame, Paris, 75004 France

**Keywords:** Expertise-based, Expertise, Systematic review, Learning, Randomised controlled trial, Trial design, Non-pharmacological interventions, Surgery

## Abstract

**Background:**

Under a conventional two-arm randomised trial design, participants are allocated to an intervention and participating health professionals are expected to deliver both interventions. However, health professionals often have differing levels of expertise in a skill-based interventions such as surgery or psychotherapy. An expertise-based approach to trial design, where health professionals only deliver an intervention in which they have expertise, has been proposed as an alternative. The aim of this project was to systematically review the use of an expertise-based trial design in the medical literature.

**Methods:**

We carried out a comprehensive search of nine databases—AMED, BIOSIS, CENTRAL, CINAHL, Cochrane Methodology Register, EMBASE, MEDLINE, Science Citation Index, and PsycINFO—from 1966 to 2012 and performed citation searches using the ISI Citation Indexes and Scopus. Studies that used an expertise-based trial design were included. Two review authors independently screened the titles and abstracts and assessed full-text reports. Data were extracted and summarised on the study characteristics, general and expertise-specific study methodology, and conduct.

**Results:**

In total, 7476 titles and abstracts were identified, leading to 43 included studies (54 articles). The vast majority (88 %) used a pure expertise-based design; three (7 %) adopted a hybrid design, and two (5 %) used a design that was unclear. Most studies compared substantially different interventions (79 %). In many cases, key information relating to the expertise-based design was absent; only 12 (28 %) reported criteria for delivering both interventions. Most studies recruited the target sample size or very close to it (median of 101, interquartile range of 94 to 118), although the target was reported for only 40 % of studies. The proportion of participants who received the allocated intervention was high (92 %, interquartile range of 82 to 99 %).

**Conclusions:**

While use of an expertise-based trial design is growing, it remains uncommon. Reporting of study methodology and, particularly, expertise-related methodology was poor. Empirical evidence provided some support for purported benefits such as high levels of recruitment and compliance with allocation. An expertise-based trial design should be considered but its value seems context-specific, particularly when interventions differ substantially or interventions are typically delivered by different health professionals.

**Electronic supplementary material:**

The online version of this article (doi:10.1186/s13063-015-0739-5) contains supplementary material, which is available to authorized users.

## Background

Under a conventional two-arm randomised controlled trial (RCT) (within-health professional) design, participants are randomly allocated to one of the two interventions and all participating health professionals are expected to deliver both interventions. However, if the interventions are skill-based, and particularly when those under evaluation differ substantially, health professionals may (for example, surgeons or therapists) may have differing levels of expertise in the interventions, conduct only one routinely, or have a preference for one over another. These potential obstacles to the conduct of an RCT have been noted particularly in surgery; many surgeons may accept the need in principle for an RCT but may struggle to reconcile their personal involvement with their surgical experience and routine practice [1]. As a result, some may be reluctant to participate in a standard, individually randomised RCT. An expertise-based approach to trial design, in which participating surgeons provide only the intervention in which they have expertise, has been proposed to overcome this problem [2]. As with the within-health professional design, a patient is randomly allocated to an intervention, the difference being that there are two sets of participating health professionals, one for each intervention, who will perform the allocated intervention. Purported benefits of this design include increased surgical participation, recruitment, and compliance with randomisation, addressing the learning curve effect as well as the desirability from the perspective of the patient [2]. Although such a design is not new [3], the profile of the design has been raised and its use appears to becoming more common. For some comparisons such as surgery versus medical management, it has become the default design. However, expertise-based designs have been criticized on a number of grounds [4], including a number of methodological considerations (for example, impact on sample size). In particular, how ‘expertise’ should be defined is uncertain but clearly is of critical importance. The optimal approach to implementation is also unclear [1, 5]. It may be that the expertise-based design is most suited to certain research questions [6–12].

Although an expertise-based design has recently received the most attention for surgical evaluation [1, 2], it has been used in other areas (for example, psychotherapy) [12]. The same issues exist for the delivery of other interventions which are substantially skill-based and in which individual practitioners may be familiar with only a particular approach or have a strong preference for one intervention over another. However, there is uncertainty regarding under which circumstances an expertise-based trial design is appropriate, how such studies are implemented in practice, and whether the purported benefits have been realised (for example, achieving recruitment and high compliance with intervention). No comprehensive review of expertise-based trials has been previously carried out. A limited search was conducted previously (160 citations) [2] but was restricted to surgery and extended only up to October 2003. Within the small number of studies identified in that search, variation in reporting and practice highlighted the need for both a comprehensive search strategy and an evaluation of differences in how such studies were carried out. For example, different terms have been used to represent the same design (for example, randomised-surgeon design). Beyond the surgical area, other terms (for example, hierarchical and nested) have been used to describe the same design [12]. Within those that might be characterised as an expertise-based design, there are variations in how ‘expertise’ was defined (for example, surgeon self-certification versus objective requirements) and how health professionals were assigned to a treatment group (health professional preference or randomisation). The aim of this review was to systematically review the use of an expertise-based trial design in the medical literature in order to improve understanding of the applicability, implementation, and implications of using such a design so as to aid the design of future trials. To achieve this, the objectives of the review were (i) to identify the contexts in which an expertise-based trial design has been used, (ii) to assess the methodological variation in expertise-based trial design implementation both in general and those aspects specifically related to the expertise-based design, and (iii) to summarise the reported experience of using such a design.

## Methods

The study protocol is available from the authors. Ethical approval was not required for this research given its focus and given that only publically available information was used.

### Inclusion criteria

RCTs of two medical interventions that used an expertise-based trial (either full or hybrid) design were included (that is, those that used a randomised allocation of participants to skill-based interventions that were delivered by non-overlapping groups of interventionalists). The following types of studies were not eligible for inclusion: those that had been published only as protocols or abstracts, trials with three or more intervention arms, and trials in which the full-text reports were published only in a non-English language journal. Systematic (or narrative) reviews of expertise-based trials were not eligible, but the included studies were assessed for inclusion. No restrictions on the age or type of participants in the trials were made.

### Search

It was anticipated, from reviewing the results of a scoping search, that it would be difficult to identify all relevant studies from searching bibliographic databases alone given the poor reporting of methods in the titles and abstracts and the paucity of specific controlled vocabulary terms in the major bibliographic databases. To address this, a range of methods were used to retrieve reports of relevant trials. The databases searched included Medical Literature Analysis and Retrieval System Online (MEDLINE), Excerpta Medica dataBASE (EMBASE), Cumulative Index to Nursing and Allied Health Literature (CINAHL), BioSciences Information Service of Biological Abstracts (BIOSIS), Cochrane Central Register of Controlled Trials (CENTRAL), Cochrane Methodology Register, Science Citation Index, The Allied and Complementary Medicine Database (AMED), and PsycINFO. In addition, cited reference searches (using Institute for Scientific Information Citation Indexes and Scopus), references of included articles, and key author searches were undertaken. If a study had been referred to as an expertise-based trial in another report but the design was unclear in the related published literature, an investigator was contacted to confirm. Searches were undertaken from 1966 onwards or, from the start of database coverage if after 1966. The MEDLINE and EMBASE multi-file search strategy, which was used to search these databases concurrently, was developed first and translated for other databases. The search strategies are provided in Additional file 1.

### Selection of studies

Two review authors (JC and CB, AE, GSM, or TB) independently screened the titles and abstracts of all reports identified by the search strategy. The full versions of articles not definitely excluded at that stage were obtained for full-text assessment, which will be carried out by two reviewers as before. Where they occurred, differences of opinion were discussed; if necessary, a blinded third review author acted as arbitrator. If appropriate, a clinical area expert was consulted. Methods articles were not included but were set aside for future reference and, if appropriate to do so, were considered in the discussion.

### Data collection and analysis

Data on the trial characteristics and context (year of publication, funding [13], clinical area, interventions under evaluation, and type of comparison), expertise-based design methodology and related reporting (type of design, reporting including stated advantages and disadvantages, interventionalist mechanism of allocation, eligibility criteria, and number), general study methodology and conduct (randomisation, blinding, and study size and compliance regarding allocation), and details on the primary outcome and statistical analyses were extracted. One reviewer (JC) carried out data extraction of included articles (using a pro forma specifically designed for the purpose). A second reviewer (AE or GM) independently assessed a random sample of 25 % of included studies. Data were summarised. Owing to the relatively small number of included studies, a planned statistical analysis to compare factors across trial types was not carried out.

## Results

**Fig. 1:**
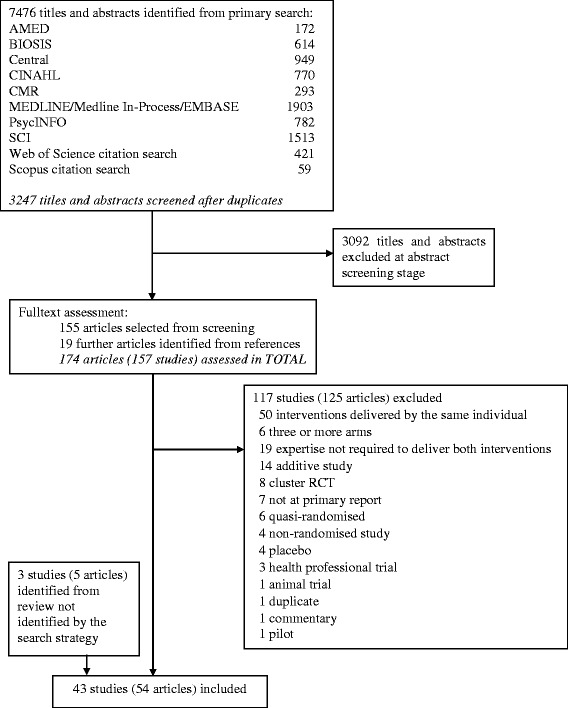
PRISMA Flow diagram. AMED, The Allied and Complementary Medicine Database; BIOSIS, BioSciences Information Service of Biological Abstracts; CENTRAL, Cochrane Central Register of Controlled Trials; CINAHL, Cumulative Index to Nursing and Allied Health Literature; CMR, Cochrane Methodology Register; EMBASE, Excerpta Medica dataBASE; MEDLINE, Medical Literature Analysis and Retrieval System Online; RCT, randomised controlled trial; SCI, Science Citation Index

In total, 7476 titles and abstracts were identified from the search of 9 databases (Fig. 1); after removal of duplicates, 3247 were screened for inclusion. Of these, 3092 were excluded and 155 articles were selected for full-text assessment. A further 19 articles were identified from the references of included studies, leading to a total of 157 studies (174 articles) that were full-text-assessed. Of these, 117 studies (125 articles) were excluded, leaving 40 studies (49 articles) that, combined with three studies (five articles) identified from a published review, led to 43 studies (54 articles) in total. The list of included studies is provided in Additional file 2. Table 1 shows the study characteristics and context of the included studies. The earliest publication was in 1982; most (69 %) were published from 2000 onwards. The vast majority received funding from public bodies in whole or part, whereas partial or full funding was received less commonly from a charity or a commercial source. The clinical areas represented varied, and mental health (26 %) and musculoskeletal (19 %) were the most common. The most common intervention types were a procedure and psychotherapy (40 and 37 % for interventions 1 and 2 for both). The comparisons overwhelmingly focussed on substantial differences between interventions (79 %).

**Table 1:** General study characteristics and context (*n* = 43 unless otherwise stated)

	Number	Percentage
Year of publication		
1980–1989	2	5
1990–1999	11	26
2000–2009	23	53
2010–2012	7	16
Funding (*n* = 28)		
Charity	3	11
Commercial	1	4
Public body	14	50
Mixed	10	37
Clinical area		
Cardiology	5	5
Emergency care	1	2
Gastroenterology	2	5
General medicine	4	9
Geriatrics	1	5
Mental health	11	26
Musculoskeletal	8	19
Neurology	3	7
Obstetrics and gynaecology	2	5
Oncology	1	2
Ophthalmology	1	2
Substance abuse	4	5
Intervention 1		
Acupuncture	1	2
Chiropractic	2	5
Clinical management (various)	1	2
Physiotherapy	3	7
Procedure	17	40
Psychotherapy	17	40
Rehabilitation management (various)	2	5
Intervention 2		
Anaesthetic	1	2
Chiropractic	1	2
Clinical management (various)	3	7
Clinical management (various)/procedure	1	2
Physiotherapy	3	7
Procedure	16	37
Psychotherapy	16	37
Rehabilitation management (varied)	2	5
Difference between interventions		
Minor	8	19
Substantial	34	79
Different type of care	1	2

Table 2 reported the methodology and reporting relating to the expertise-based trial design. The vast majority (88 %) used a pure expertise-based design in which health professionals deliver only one of the two interventions; only three (7 %) used a hybrid designin which some health professionals could deliver both. For two studies the design that was unclear (5 %). Ten used terminology to refer to the design, and six different names were used. The level of detail reported in the abstract varied from stating the design name in six (14 %) studies to giving no details in 15 (35 %). A variety of advantages of using an expert-based design were stated: the most common were ensuring a balance of health professionals in terms of interest and commitment (9 %), addressing learning (7 %), ensuring that the intervention is delivered by someone with expertise in order to avoid criticism (5 %), and reducing cross-over (5 %). Fewer disadvantages were stated; the more common were that intervention deliverers were not representative of clinical practice or may not be balanced between groups unless selected (9 %) and that the delivery of interventions may vary between groups beyond the allocated intervention (5 %). Under half reported the method of allocation of health professoinal to intervention group; eight (19 %) interestingly used randomisation, five (12 %) followed usual practice, four followed interventionalist preference (9 %), and in one case (2 %) the type of health professional was defined as part of the research question. Criteria for delivering an intervention varied between groups and involved one or more of health professional training/grades, years of experience in and/or number of cases of delivering the intervention, prior training and/or supervision in using the intervention, specified outcome performance level, recommendation by colleague, experience of working with patient group, and willingness to learn a new procedure. Only 12 (28 %) stated the criteria for delivering both interventions. A substantial number (21,49 %) did not give the number of health professionals delivering the interventions for both groups. The number of health professionals was similar for the two arms (medians of 6 and 5, respectively).

**Table 2:** Expertise-based design methodology and related reporting (*n* = 43 unless otherwise stated)

	Number	Percentage
Expertise-based design type		
Pure	38	88
Hybrid	3	7
Unclear	2	5
Name used (*n* = 24)		
Expertise-based	2	8
Double randomisation	2	8
Randomised to surgeon	2	8
Non-randomised surgeon design	1	4
Randomised-surgeon	1	4
Surgeon-randomised	2	8
None	15	63
Reporting of expertise-based design in abstract		
Design name	6	14
Deliverers of interventions stated to be different	9	21
Details regarding health professionals delivering one intervention	7	16
Details regarding health professional delivering both interventions	6	14
No details	15	35
Reported advantages (*n* = 20)		
Ensuring intervention was delivered by someone with expertise to avoid criticism of the study	2	5
Balance of health professionals (e.g., interest, commitment, and prior knowledge of intervention)	4	9
Randomisation of health professional ‘consistent with efficacy trial’	1	2
Following preference will reduce non-compliance	1	2
Using randomisation of health professional strengthens generalisability of findings	1	2
Eliminates learning of the intervention	3	7
Eliminates ethical concerns with intervention deliverer not doing what they would do outside of the trial	1	2
Delivery of intervention maximised (and may reduce adverse events)	1	2
Ensures experience in control group	1	2
Reduces cross-over between group compared with conventional study	2	5
Avoid non-compliance with allocation because of non-familiarity	1	2
Health professionals delivering their preferred intervention	1	2
Following usual practice reduces non-compliance with allocation	1	2
Reported disadvantages (*n* = 9)		
Health professionals delivering interventions may not be representative of practice	1	2
Health professionals delivering interventions may not be balanced (e.g., motivation and prior experience) unless selected	4	9
Delivery may vary in other ways between groups because of different health professionals delivering the interventions	2	5
Disagreement between recruiter and health professional delivering the intervention regarding eligibility led to the intervention not being performed in some cases	1	2
Addition of new intervention and deliverer may create expectation bias	1	2
Allocation of intervention deliverers		
Randomised	8	19
Usual practice	5	12
Preference	4	9
Defined by research question	1	2
Not stated	25	58
Criteria for delivering intervention 1		
Number of prior cases	2	4
Number of years of experience and prior cases	1	2
Number of years of experience and training in intervention	1	2
‘Qualified’ intervention deliverer	1	2
Training of therapy and group supervision	1	2
Profession qualification	3	6
Prior training and experience of intervention	1	2
Trained in delivering intervention	4	8
Recommendation by colleagues as expert	1	2
Experience of working with patient group	1	2
Willingness to learn new intervention	1	2
Without prior experience of intervention (training then provided)	1	2
None (training/supervision provided as part of the study)	3	7
Not stated	22	51
Criteria for delivering intervention 2		
Number of years of experience and specific outcome levels to be achieved	1	2
Years of experience	1	2
Number of years of experience and prior cases	1	2
Recommendation by colleagues as expert	1	2
Experience of working with patient group	1	2
Professional qualification	2	4
Preference and no training in alternative intervention	1	2
Willingness to learn new intervention	1	2
Interest in patient group	1	2
None (trained as part of the study)	2	5
None stated	31	72
Criteria provided for both intervention 1 and 2 deliverers	12	28
Number of health professionals delivering intervention 1		
Reported	30	69
Median (interquartile range), range	6 (2–12), 1–58	
Number of health professionals delivering intervention 2		
Fully reported	23	53
Median (interquartile range), range	5 (2–19), 1–63	

Details regarding general study methodology and conduct are shown in Table 3. Reporting of participant randomisation methodology was adequate for the majority of studies (58 and 60 % for sequence generation and allocation concealment, respectively). Blinding of the outcome assessment was known to be performed in 12 (28 %) studies, but blinding of participants was known to be performed in only one (2 %). Most studies were single-centre RCTs (median of 1, interquartile range (IQR) of 1 to 5). Study size was typically moderate (111, IQR of 62 to 226). Most studies recruited the target number or very close to it (101, IQR of 94 to 118), although the target size was reported for only 40 % of studies. The proportion of participants who received the allocated intervention was high (92 %, IQR of 82 to 99 %). For those that reported this aspect, occurrences of cross-over between interventions or receiving a ‘third’ (non-trial) intervention were rare. Table 4 reports details on the primary outcome. The most common outcomes were type of disease and specific quality of life (29 %) followed by other patient-reported outcomes and process measure (17 % for both). The number of observations available to analyse was poorly reported (26 studies, or 60 %). Details regarding the statistical analysis carried out are provided in Table 5. Almost all studies (98 %) carried out an analysis by randomised groups. Eight (19 %) studies had a compliance analysis, and only two (5 %) adjusted for clustering of outcome by interventionalists.

**Table 3:** Study methodology and conduct (*n* = 43 unless otherwise stated)

	Number	Percentage
Intervention deliverers–randomisation sequence generation		
Adequate	1	10
Inadequate	0	0
Unclear	9	90
Intervention deliverers–randomisation allocation concealment		
Adequate	1	10
Inadequate	0	0
Unclear	9	90
Participants–randomisation sequence generation		
Adequate	25	58
Inadequate	0	0
Unclear	18	42
Participants–randomisation allocation concealment		
Adequate	26	60
Inadequate	2	5
Unclear	15	35
Blinding–primary outcome assessment		
Yes	12	28
No	27	63
Unclear	4	9
Blinding–participants		
Yes	1	2
No	34	79
Unclear	8	19
Number of centres		
Reported	42	98
Median (IQR)	1	(1–5)
Study size		
Reported	42	98
Median (IQR)	111	(62–226)
Percentage of target size recruited		
Reported target size	17	40
Median (IQR)	101	(94–118)
Percentage received full intervention		
Reported	31	74
Median (IQR)	92	(82–99)
Cross-over to other study intervention		
Reported	18	42
Median (IQR)	0	(0–2)
“Third” intervention		
Reported	15	35
Median (IQR)	0	(0–0)

*IQR* interquartile range

**Table 4:** Primary outcome(s) (*n* = 43 unless otherwise stated)

	Number	Percentage
Primary outcome		
Number of primary outcomes		
None	17	40
1	15	35
2	5	12
3	3	7
4	2	5
5	0	0
6	1	2
Type of outcome (*n* = 48)		
Pain	4	8
Generic quality of life	2	4
Disease-specific quality of life	14	29
Treatment success	4	8
Other patient-reported outcome	8	17
Mortality, composite including mortality	2	4
Process measure	8	17
Functional measure	6	13
Valid observations (*n* = 26)^a^, median (interquartile range), range	127 (78–435), 24–4752	

^a^For studies with more than one primary outcome, the maximum available number of observations was used

**Table 5:** Statistical analysis methodology (*n* = 43 unless otherwise stated)

	Number	Percentage
Analysis groups according to randomised groups		
Yes	42	98
No	1	2
Unclear	0	0
Analysis adjusting for non-compliance carried out		
Yes	8	19
No	35	81
Unclear	0	0
Compliance analysis type		
As treated groups	3	7
Intervention completer subset analysis (full compliance)	2	5
Intervention completer subset analysis (partial and full compliance)	2	5
Randomisation-based analysis (method unclear)	1	2
N/A	35	81
Analysis adjusting for clustering^a^		
Yes	2^b^	5
No	41	95
Unclear	0	0

*N*/*A* not available

^a^Analyses carried out were mixed-effect models: one with two levels (intervention deliverer and) and one with three (site, intervention deliverer, and participant). ^b^In one study, the sample size was adjusted to account for clustering by using an intracluster correlation coefficient (ICC) of 0.02; for the other study the observed ICC of the higher level(s) was reported for the two primary outcomes (retention and engagement) under a two level (therapist and participant) and a 3 level model (site, therapist and participant) as 0.180 and 0.22 and 0.099 and 0.110 respectively

## Discussion

This systematic review identified from across the medical literature a number of RCTs that used an expertise-based trial design. An expertise-based design differs from the conventional (within-health professional) design by allowing different groups of health professionals to deliverer the interventions under evaluation as opposed to requiring participating health professionals to deliver both of (or all, where there are three or more) the interventions. It has been suggested that the design is able to address [2, 3] some of the challenges of conducting an RCT of skill-based interventions which a conventional design is not. Although the design has been in use for over 30 years and use has been growing in recent years, it remains uncommon.

Previous limited reviews [2, 14] had identified fewer than 10 studies using an expertise-based design and focussed only on surgery. Uniquely, this review had a full and comprehensive systematic search with information on the context of the study, the methodology of the trial (both expertise-related and generic), and the reported views of the trialists collected and summarised. It shows that the design has been used beyond surgical interventions where its use has been previously proposed [2, 3, 15] and covers many clinical areas and evaluations of non-pharmacological interventions. The potential value over a conventional design appears to be case-specific and is clear in two main circumstances. One is where two skill-based interventions that are substantially different from one another are to be compared (for example, cognitive behavioural therapy and limited professional support); this is the situation where they have most commonly been used. Another setting where they have been used and seem particularly appropriate (or perhaps natural to use) is when the interventions are typically delivered by another type of health professional (for example, a comparison of coronary artery bypass graft and stenting for the treatment of coronary artery disease where they are delivered by a surgeon and a cardiologist respectively, as is the case in some countries). Two surveys of preferences regarding trial design showed differing levels of support for an expertise-based design, suggesting that it merits consideration with regard to the specific context in which it would be used [6, 9].

Reporting of the use of the expertise-based design methodology in the abstract was highly variable, and insufficient details regarding key methodological features of expertise-based trials were provided. Around half failed to provide details on the interventionalists delivering the interventions, a key piece of information for interpreting and assessing the applicability of the findings. Furthermore, we identified the use of a hybrid approach in a small number of studies, although it is possible that more studies adopted this design, but this information was not provided in the trial reports [5, 16]. Expertise-based trials were typically single-centre studies of moderate size with around 10 interventionalists involved in the study with a patient-reported primary outcome. Two of the main purported benefits appeared to be supported by the empirical evidence. Achievement of the target recruitment was very high, although the target size was often not reported, leading to some uncertainty regarding this finding. However, compliance with the allocated intervention was generally very high (median of at least 90 %), and occurrences of cross-over between intervention groups or receiving a third non-trial intervention were rare.

Reporting of general study methodology such as randomisation was suboptimal, as has been repeatedly shown for RCTs of both pharmacological and non-pharmacological interventions [17]. Blinding of the primary outcome assessment and of participation was not typically performed, perhaps reflecting the nature of the trial comparison and the types of interventions under evaluation. Studies appeared to adopt a more pragmatic approach to study design [2]. Only two studies adjusted the analysis for clustering of outcome by interventionalist, even though failure to do this could lead to an overly precise estimate, and no studies provided justification for why this was not done [7, 18].

A number of potential advantages have been proposed to support the use of an expertise-based trial design, including addressing the learning curve and possible differences in expertise among health professionals between interventions (sometimes referred to as differential expertise bias, which is particularly likely if a new intervention is compared with an older one), ethical concerns of intervention deliverers about performing a procedure they are not experienced or comfortable with, reassurance for patients that their intervention will be delivered by someone with appropriate ‘expertise’, improved compliance, avoidance of systematic bias introduced unconsciously if a preference for one intervention exists, and increased participation of interventionalists [2, 3]. However, a number of potential drawbacks have also been noted: the feasibility of two different interventionalists being available, imbalances between interventionalist groups in terms of ability and experience, the need for third-party recruitment, and reduced applicability of the study results [2, 4, 6, 15, 19]. This review generally reinforced the proposed advantages and disadvantages but highlighted that some of the advantages are conditional on the implementation of the expertise-based design regarding definition of “expertise” (for example, elimination learning). Additional disadvantages were identified, such as the possibility of an expectation bias being created if a new intervention is delivered by a different individual [20], having different individuals recruiting and delivering the intervention which can lead to disagreement regarding suitability for receiving the intervention [21], and the possibility that the intervention may differ in ways beyond the treatment allocation [20, 22].

There were a number of limitations to this review. Owing to the inconsistency of reporting highlighted in included studies, it seems likely that some eligible expertise-based trials were not identified, as reference to this aspect of the design was not made in the title or abstracts or possibly anywhere in the text. Inclusion was restricted to two-arm individually randomised parallel group trials, although a form of expertise-based design could be used in other settings (for example, three-arm or cluster RCT). Additionally, the search was conducted only up to 2012. Owing to the small numbers of included studies, it was not possible to formally compare subtypes of trials as planned. Finally, no formal comparison has been made with conventional (within-health professional) trials.

This review expands the current understanding of expertise-based trials. Further work exploring the view and experience of health professionals and those involved in trial design should be carried out to further increase understanding of the decision-making process regarding design choice. We recommend improved and more consistent reporting, and reports should state explicitly who delivered the interventions; the term ‘expertise-based’ could be used in the title or abstract of trial reports with explicit details regarding the number of interventionalists in each arm with any criteria for eligibility reported in the trial report.

## Conclusions

Although the use of an expertise-based trial design is growing, it remains uncommon. An expertise-based trial design is an option that should be considered by trials, particularly in light of the high level of recruitment to target and compliance with allocation observed [23]. Its value appears to be context-specific, particularly where interventions differ substantially or interventions are typically delivered by different health professionals. Reporting of expertise-based trials is suboptimal in providing details relating to the expertise design and requires improvement.

## Additional files

Additional file 1:
**‘Additional file 1–Search Strategies.docx’, Search strategies, Database search strategies.**
Additional file 2:
**‘Additional file 1–Search Strategies.docx’, Included studies, List of included studies.**

## Abbreviations

EMBASE: Excerpta Medica dataBASE
IQR: interquartile range
MEDLINE: Medical Literature Analysis and Retrieval System Online
RCT: randomised controlled trial
