# Supplementary material for: A systematic review of the use of an expertise-based randomised controlled trial design
Source: Trials. 2015 May 30;16:241. doi: 10.1186/s13063-015-0739-5 (PMC4468810; doi:10.1186/s13063-015-0739-5)
Supplement: Additional file 1: — ‘Additional file 1–Search Strategies.docx’, Search strategies, Database search strategies. [file 13063_2015_739_MOESM1_ESM.docx]

**Search strategies**

**Database: Embase <1980 to 2012 Week 34>, Ovid MEDLINE(R) <1946 to August Week 3 2012>, Ovid MEDLINE(R) In-Process & Other Non-Indexed Citations <August 27, 2012>**

**OVID Multi-file Search URL:** [**https://shibboleth.ovid.com/**](https://shibboleth.ovid.com/)

1 (surgeon$ adj5 (random$ or assign$ or allocat$ or nest$ or effect)).tw.

2 (therapist$ adj5 (random$ or assign$ or allocat$ or nest$ or effect)).tw.

3 (surgeon$ adj5 (random$ or assign$ or allocat$ or nest$ or effect)).tw.

4 (physiotherapist$ adj5 (random$ or assign$ or allocat$ or nest$ or effect)).tw

5 (psychotherapist$ adj5 (random$ or assign$ or allocat$ or nest$ or effect)).tw.

6 (radiotherapist$ adj5 (random$ or assign$ or allocat$ or nest$ or effect)).tw.

7 (chiropractor$ adj5 (random$ or assign$ or allocat$ or nest$ or effect)).tw. (7

8 (expertise adj5 based).tw.

9 equipoise.tw. (

10 ((nested or hierarchical) adj3 (design or trial? or random$)).tw.

11 or/1-10 (8966)

12 exp *controlled clinical trials as topic/ use mesz

13 randomized controlled trial.pt.

14 controlled clinical trial.pt.

15 (randomi?ed or randomly).tw.

16 exp *controlled clinical trial/ use emez

17 *randomization/ use emez

18 or/12-17

19 11 and 18

20 remove duplicates from 19

21 20 and conference abstract.pt.

22 20 not 21

**Database: AMED (Allied and Complementary Medicine) <1985 to August 2012>**

**OVID URL: https://shibboleth.ovid.com/**

1 (surgeon$ adj5 (random$ or assign$ or allocat$ or nest$ or effect)).tw.

2 (therapist$ adj5 (random$ or assign$ or allocat$ or nest$ or effect)).tw.

3 (surgeon$ adj5 (random$ or assign$ or allocat$ or nest$ or effect)).tw.

4 (physiotherapist$ adj5 (random$ or assign$ or allocat$ or nest$ or effect)).tw.

5 (psychotherapist$ adj5 (random$ or assign$ or allocat$ or nest$ or effect)).tw.

6 (radiotherapist$ adj5 (random$ or assign$ or allocat$ or nest$ or effect)).tw.

7 (chiropractor$ adj5 (random$ or assign$ or allocat$ or nest$ or effect)).tw.

8 (expertise adj5 based).tw.

9 equipoise.tw.

10 ((nested or hierarchical) adj3 (design or trial? or random$)).tw.

11 or/1-10

12 randomized controlled trial.pt

13 controlled clinical trial.pt.

14 (randomi?ed or randomly).tw.

15 or/12-14

16 11 and 15

**Cochrane Central Register of Controlled Trials (Central)**

**Issue 8 of 12, Aug 2012**

**The Cochrane Library** **URL:** [**http://www3.interscience.wiley.com/**](http://www3.interscience.wiley.com/)

#1 (surgeon* NEAR/5 (random* or assign* or allocat* or nest* or effect))

#2 (therapist* NEAR/5 (random* or assign* or allocat* or nest* or effect))

#3 (physiotherapist* NEAR/5 (random* or assign* or allocat* or nest* or effect))

#4 (radiotherapist* NEAR/5 (random* or assign* or allocat* or nest* or effect))

#5 (chiropractor* NEAR/5 (random* or assign* or allocat* or nest* or effect))

#6 (expertise NEAR/5 based) or (equipoise): in Trials 23 edit delete

#7 (hierarchical NEAR/3 (design or trial* or random*)) or (nested NEAR/3 (design or trial* or random*))

#8 (#1 OR #2 OR #3 OR #4 OR #5 OR #6 OR #7)

**Cochrane Methodology Register**

**Issue 3 2012**

**The Cochrane Library** **URL:** [**http://www3.interscience.wiley.com/**](http://www3.interscience.wiley.com/)

#1 (expertise NEAR/5 based) or (equipoise)

#2 (hierarchical NEAR/3 (design or trial* or random*)) or (nested NEAR/3 (design or trial* or random*))

#3 "bias in trials" AND "random allocation":kw

#1 or #2 or #3

**Science Citation Index 1966 - 29^th^ August 2012 Biosis 1966 - 30^th^ August 2012**

**ISI Web of Knowledge URL:** [**http://wok.mimas.ac.uk/**](http://wok.mimas.ac.uk/)

# 1 (TS=(surgeon$ NEAR/5 (random* or assign* or allocat* or nest* or effect)).)

# 2 (TS=(therapist$ NEAR/5 (random* or assign* or allocat* or nest* or effect)))

# 3 (TS=(physiotherapist$ NEAR/5 (random* or assign* or allocat* or nest* or effect)))

# 4 (TS=(psychotherapist$ NEAR/5 (random* or assign* or allocat* or nest* or effect)))

# 5 (TS=(radiotherapist$ NEAR/5 (random* or assign* or allocat* or nest* or effect)))

# 6 (TS=(chiropractor$ NEAR/5 (random* or assign* or allocat* or nest* or effect)))

# 7 (TS=(expertise NEAR/5 based))

# 8 (TS=equipoise)

# 9 (TS= ((nested or hierarchical) NEAR/3 (design or trial$ or random*)))

# 10 #9 OR #8 OR #7 OR #6 OR #5 OR #4 OR #3 OR #2 OR #1

# 11 (TS=randomi$ed)

# 12 (TS=randomly)

# 13 #12 OR #11

# 14 #13 AND #10 AND Document Types=(Article)

**Cumulative Index of Nursing and Allied Health Literature (CINAHL) 1981 - 30^th^ August 2012**

**EBSCO URL:** [**http://web.ebscohost.com/**](http://web.ebscohost.com/)

S1 TX ( (surgeon* N5 (random* or assign* or allocat* or nest* or effect)) ) OR TX ( (nurse* N5 (random* or assign* or allocat* or nest* or effect)) ) OR TX ( (chiropractor* N5 (random* or assign* or allocat* or nest* or effect)) )

S2 TX ( (therapist* N5 (random* or assign* or allocat* or nest* or effect)) ) OR TX ( (physiotherapist* N5 (random* or assign* or allocat* or nest* or effect)) ) OR TX ( (psychotherapist* N5 (random* or assign* or allocat* or nest* or effect)) ) AND ( (radiootherapist* N5 (random* or assign* or allocat* or nest* or effect)) )

S3 TX (expertise N5 based) OR TX equipoise OR TX ( (nested N3 (design or trial* or random*)). ) OR TX ( (hierarchical N3 (design or trial* or random*)). )

S4 S1 or S2 or S3

S5 TX randomi?ed or randomly

S6 (MH "Randomized Controlled Trials")

S7 S4 and S5

S8 S4 and S6

S9 S7 or S8

S10 S7 or S8 Limiters - Exclude MEDLINE records

**PsycINFO 1966 - 29^th^ August 2013**

**EBSCO URL: http://web.ebscohost.com/**

S1 TX ( (psychologist* N5 (random* or assign* or allocat* or nest* or effect)) )

S2 TX ( (therapist* N5 (random* or assign* or allocat* or nest* or effect)) ) OR TX ( (psychotherapist* N5 (random* or assign* or allocat* or nest* or effect))

S3 TX (expertise N5 based) OR TX equipoise OR TX ( (nested N3 (design or trial* or random*)). ) OR TX ( (hierarchical N3 (design or trial* or random*)). )

S4 s1 or s2 or s3

S5 TX (randomi?ed N3 trial*)

S6 s4 and s5
