# Supplementary material for: A systematic review of the use of an expertise-based randomised controlled trial design
Source: Trials. 2015 May 30;16:241. doi: 10.1186/s13063-015-0739-5 (PMC4468810; doi:10.1186/s13063-015-0739-5)
Supplement: Additional file 2: — ‘Additional file 1–Search Strategies.docx’, Included studies, List of included studies. [file 13063_2015_739_MOESM2_ESM.docx]

**Included studies n=43**

1 Coronary angioplasty versus coronary artery bypass surgery: the Randomized Intervention Treatment of Angina (RITA) trial. Lancet 1993;341:573-80.

2 First-year results of CABRI (Coronary Angioplasty versus Bypass Revascularisation Investigation). CABRI Trial Participants. Lancet 1995;346:1179-84.

3 Comparison of coronary bypass surgery with angioplasty in patients with multivessel disease. The Bypass Angioplasty Revascularization Investigation (BARI) Investigators. N Engl J Med 1996;335 :217-25.

4 Addis ME, Hatgis C, Krasnow AD, Jacob K, Bourne L, Mansfield A. Effectiveness of cognitive-behavioral treatment for panic disorder versus treatment as usual in a managed care setting. J Consult Clin Psychol 2004;72:625-35.

5 Alobaid A, Harvey EJ, Elder GM, Lander P, Guy P, Reindl R. Minimally invasive dynamic hip screw. Prospective randomized trial of two techniques of insertion of a standard dynamic fixation device. J Orthop Trauma 2007;18:7-212.

6 Ball SA, Martino S, Nich C, Frankforter TL, Van HD, Crits-Christoph P et al. Site matters: multisite randomized trial of motivational enhancement therapy in community drug abuse clinics. J Consult Clin Psychol 2007;75:556-67.

7 Barrington J, Prior M, Richardson M, Allen K. Effectiveness of CBT versus standard treatment for childhood anxiety disorders in a community clinic setting. Behaviour Change 2005; 22:29-43.

8 Baskett JJ, Broad JB, Reekie G, Hocking C, Green G. Shared responsibility for ongoing rehabilitation: A new approach to home-based therapy after stroke. Clin Rehabil 1999;13:23-33.

9 Bateman A, Fonagy P. Randomized Controlled Trial of Outpatient Mentalization-Based Treatment Versus Structured Clinical Management for Borderline Personality Disorder. Am J Psychiatry 2009;166:1355-64.

10 Bedics JD, Atkins DC, Comtois KA, Linehan MM. Treatment differences in the therapeutic relationship and introject during a 2-year randomized controlled trial of dialectical behavior therapy versus nonbehavioral psychotherapy experts for borderline personality disorder. J Consult Clin Psychol 2012;80:66-77.

11 Borduin CM, Schaeffer CM, Heiblum N. A randomized clinical trial of multisystemic therapy with juvenile sexual offenders: Effects on youth social ecology and criminal activity. J Consult Clin Psychol 2009;77:26-37.

12 Chen PT, Sung CS, Wang CC, Chan KH, Chang WK, Hsu WH. Experience of anesthesiologists with percutaneous nonangiographic venous access. J Clin Anesth 2007;19:609-15.

13 David R, Enderby P, Bainton D. Treatment of acquired aphasia: Speech therapists and volunteers compared. J Neurol, Neurosurg Psychiatry 1982;45:957-61.

14 Doering S, Horz S, Rentrop M, Fischer-Kern M, Schuster P, Benecke C et al. Transference-focused psychotherapy v. treatment by community psychotherapists for borderline personality disorder: Randomised controlled trial. Br J Psychiatry 2006;#2010. :389-95.

15 Esposito-Smythers C, Spirito A, Kahler CW, Hunt J, Monti P. Treatment of co-occurring substance abuse and suicidality among adolescents: A randomized trial. J Consult Clin Psychol 2011;79:728-39.

16 Grant AM, Wileman SM, Ramsay CR, Mowat NA, Krukowski ZH, Heading RC et al. Minimal access surgery compared with medical management for chronic gastro-oesophageal reflux disease: UK collaborative randomised trial. BMJ 2009;338:81-3.

17 Gudavalli MR, Cambron JA, McGregor M, Jedlicka J, Keenum M, Ghanayem AJ et al. A randomized clinical trial and subgroup analysis to compare flexion-distraction with active exercise for chronic low back pain. Eur Spine J 2006;15:1070-82.

18 Henggeler SW, Melton GB, Brondino MJ, Scherer DG, Hanley JH. Multisystemic therapy with violent and chronic juvenile offenders and their families: The role of treatment fidelity in successful dissemination. J Consult Clin Psychol 1997;65:821-33.

19 Johnson DP, Penn DL, Bauer DJ, Meyer P, Evans E. Predictors of the therapeutc alliance in group therapy for individuals with treatment-resistant auditory hallucinations. Br J Clin Psychol 2008;47:171-83.

20 Jouriles EN, McDonald R, Rosenfield D, Norwood WD, Spiller L, Stephens N et al. Improving parenting in families referred for child maltreatment: A randomized controlled trial examining effects of Project Support. J FamPsychol 2010;24:328-38.

21 Kajiyama T, Hajiro K, Sakai M, Inoue K, Konishi Y, Takakuwa H et al. Endoscopic resection of gastrointestinal submucosal lesions: A comparison between strip biopsy and aspiration lumpectomy. Gastrointest Endosc 1996;44:404-10.

22 Knebel P, Lopez-Benitez R, Fischer L, Radeleff BA, Stampfl U, Bruckner T et al. Insertion of totally implantable venous access devices: An expertise-based, randomized, controlled trial (NCT00600444). Ann Surg 2011;253:1111-7.

23 Lamy A, Devereaux PJ, Prabhakaran D, Hu SS, Piegas LS, Straka Z et al. Rationale and design of The Coronary Artery Bypass Grafting Surgery Off or On Pump Revascularization Study: A large international randomized trial in cardiac surgery. Am Heart J 2012;163:1-6.

24 Machler HE, Bergmann P, Anelli-Monti M, Dacar D, Rehak P, Knez I et al. Minimally invasive versus conventional aortic valve operations: A prospective study in 120 patients. Ann Thorac Surg 1999;67:1001-5.

25 Moore J, Shank JR, Morgan SJ, Smith WR. Syndesmosis fixation: A comparison of three and four cortices of screw fixation without hardware removal. Foot Ankle Int 2006;27:567-72.

26 Mourits MJE, Bijen CB, Arts HJ, ter Brugge HG, van der Sijde R, Paulsen L et al. Safety of laparoscopy versus laparotomy in early-stage endometrial cancer: a randomised trial. Lancet Oncol 2010;11:763-71.

27 Petersen Ttpkd, Larsen K, Nordsteen J, Olsen S, Fournier G, Jacobsen S. The McKenzie Method Compared With Manipulation When Used Adjunctive to Information and Advice in Low Back Pain Patients Presenting With Centralization or Peripheralization A Randomized Controlled Trial. Spine 2011;36:1999-2010.

28 Phillips WA, Schwartz HS, Keller CS, Woodward HR, Rudd WS, Spiegel PG et al. A prospective, randomized study of the management of severe ankle fractures. J Bone Joint Surg Am 1985;67:67-78.

29 Richardson B, Shepstone L, Poland F, Mugford M, Finlayson B, Clemence N. Randomised controlled trial and cost consequences study comparing initial physiotherapy assessment and management with routine practice for selected patients in an accident and emergency department of an acute hospital. Emerg Med J 2005;22:87-92.

30 Robbins MS, Feaster DJ, Horigian VE, Rohrbaugh M, Shoham V, Bachrach K et al. Brief strategic family therapy versus treatment as usual: Results of a multisite randomized trial for substance using adolescents. J Consult Clin Psychol 2011;79:713-27.

31 Ruit S, Tabin G, Chang D, Bajracharya L, Kline DC, Richheimer W et al. A prospective randomized clinical trial of phacoemulsification vs manual sutureless small-incision extracapsular cataract surgery in Nepal. Am J Ophthalmol 2007;143:32-8.

32 Scogin F, Morthland M, Kaufman A, Burgio L, Chaplin W, Kong G. Improving quality of life in diverse rural older adults: A randomized trial of a psychological treatment. Psychol Aging 2007;22:657-65.

33 Southam-Gerow MA, Weisz JR, Chu BC, McLeod BD, Gordis EB, Connor-Smith JK. Does cognitive behavioral therapy for youth anxiety outperform usual care in community clinics? An initial effectiveness test. J Am Acad Child Adolesc Psychiatry 2010;49:1043-52.

34 Tate JJT, Dawson JW, Chung SCS, Lau WY, Li AKC. Laparoscopic versus open appendicectomy: Prospective randomised trial. Lancet 1993;342:633-7.

35 Taylor JD, Fletcher JP, Tiarks J. Impact of physical therapist-directed exercise counseling combined with, fitness center-based exercise training on muscular strength and exercise, capacity in people with type 2 diabetes: A randomized clinical trial. Phys Ther 2009;89:884-92.

36 Ursini T, Tontodonati M, Manzoli L, Polilli E, Rebuzzi C, Congedo G et al. Acupuncture for the treatment of severe acute pain in herpes zoster: results of a nested, open-label, randomized trial in the VZV Pain Study. BMC Complement Altern Med 2011;11:46.

37 van EB, Staal JB, van A, de B, van d. No difference between postural exercises and strength and fitness exercises for early, non-specific, work-related upper limb disorders in visual display unit workers: A randomised trial. Austral J Physiotherapy 2008;54:95-101.

38 Weisz JR, Southam-Gerow MA, Gordis EB, Connor-Smith JK, Chu BC, Langer DA et al. Cognitive-Behavioral Therapy Versus Usual Clinical Care for Youth Depression: An Initial Test of Transportability to Community Clinics and Clinicians. J Consult Clin Psychol 2009;77:383-96.

39 Widen H, Von K, Kostulas V, Holm M, Widsell G, Tegler H et al. A randomized controlled trial of rehabilitation at home after stroke in southwest Stockholm. Stroke 1998;29:591-7.

40 Wihlborg O. Fixation of femoral neck fractures: A four-flanged nail versus threaded pins in 200 cases. Acta Orthop Scand 1990;61:415-8.

41 Wilkey A, Gregory M, Byfield D, McCarthy PW. A comparison between chiropractic management and pain clinic management for chronic low-back pain in a national health service outpatient clinic. J Altern Complement Med 2008;14:465-73.

42 Winhusen T, Kropp F, Babcock D, Hague D, Erickson SJ, Renz C et al. Motivational enhancement therapy to improve treatment utilization and outcome in pregnant substance users. J Subst Abuse Treat 2008;35:161-73.

43 Wyrsch B, McFerran MA, McAndrew M, Limbird TJ, Harper MC, Johnson KD et al. Operative treatment of fractures of the tibial plafond: A randomized prospective study. J Bone Joint Surg A 1996;78:1646-57.
